# Evidence of Stem Cells Mobilization in the Blood of Patients with Pancreatitis: A Potential Link with Disease Severity

**DOI:** 10.1155/2022/5395248

**Published:** 2022-07-08

**Authors:** Krzysztof Dąbkowski, Anna Łabędź-Masłowska, Barbara Dołęgowska, Krzysztof Safranow, Marta Budkowska, Ewa Zuba-Surma, Teresa Starzyńska

**Affiliations:** ^1^Department of Gastroenterology, Faculty of Medicine and Dentistry, Pomeranian Medical University, Unii Lubelskiej 1, 71-252 Szczecin, Poland; ^2^Department of Cell Biology, Faculty of Biochemistry, Biophysics and Biotechnology, Jagiellonian University, Gronostajowa 7, 30-387 Krakow, Poland; ^3^Department of Microbiology, Immunology and Laboratory Medicine, Faculty of Medicine and Dentistry, Aleja Powstańców Wielkopolskich 72, 70-111 Szczecin, Poland; ^4^Department of Biochemistry and Medical Chemistry, Faculty of Medicine and Dentistry Pomeranian Medical University, Aleja Powstańców Wielkopolskich 72, 70-111 Szczecin, Poland; ^5^Department of Medical Analytics, Faculty of Medicine and Dentistry Pomeranian Medical University, Aleja Powstańców Wielkopolskich 72, 70-111 Szczecin, Poland

## Abstract

A growing number of studies indicate the potential involvement of various populations of bone marrow-derived stem cells (BMSCs) in tissue repair. However, the mobilization of BMSCs to the peripheral blood (PB) in acute and chronic pancreatitis (AP and CP) has not been investigated. A total of 78 patients were assigned into AP, CP, and healthy control groups in this study. Using flow cytometry, we found that VSELs, EPCs, and CD133^+^SCs were mobilized to the PB of patients with both AP and CP. Interestingly, AP and CP patients exhibited lower absolute number of circulating MSCs in the PB compared to healthy individuals. SC mobilization to the PB was more evident in patients with AP than CP and in patients with moderate/severe AP than mild AP. Using ELISA, we found a significantly increased HGF concentration in the PB of patients with AP and SDF1*α* in the PB of patients with CP. We noted a significant positive correlation between SDF1*α* concentration and the mobilized population of CD133^+^SCs in AP and between C5a and the mobilized population of VSELs moderate/severe AP. Thus, bone marrow-derived SCs may play a role in the regeneration of pancreatic tissue in both AP and CP, and mobilization of VSELs to the PB depends on the severity of AP.

## 1. Introduction

The bone marrow (BM) is a complex organ containing many different stem cell (SC) populations, such as hematopoietic stem cells (HSCs) and nonhematopoietic stem cells, including endothelial progenitor cells (EPCs), mesenchymal stem/stromal cells (MSCs), multipotential adult progenitor cells, pluripotent stem cells (PSCs), and tissue committed stem cells [1, 2]. BM-derived nonhematopoietic cells are released (mobilized) from the BM into the peripheral blood (PB) during tissue injury [3]. The phenomenon of BM-derived stem cell (BMSC) mobilization to the PB was previously described in different inflammatory disorders and diseases related to tissue injury and regeneration processes accompanying, for example, skin burns [4], sepsis [5], septic shock [6], Crohn's disease [7], leg ischemia [8], myocardial infarction [9], or stroke [10]. BMSCs have been postulated to act as “paramedics,” migrating from the BM into the PB, and subsequently to the inflammation site, where they may contribute in the process of tissue healing and regeneration [11]. By this time, the circulation of different populations of BMSCs in the PB during the course of pancreatic disorders in humans was only examined by our group in patients with pancreatic ductal adenocarcinoma (PDAC) [12]. The phenomenon of the circulation of distinct populations of BMSCs in the blood of patients suffering from pancreatic inflammatory diseases has not yet been investigated. In case of pancreatic carcinogenesis, BMSCs are suspected to play a pivotal role in the constitution of the cancer microenvironment [13]. BMSCs may differentiate into multiple cell types, forming crucial elements of the cancer stroma, but do not differentiate into cells of the cancer epithelium as has was observed during stomach cancer [14, 15]. Moreover, BMSCs may interact with neoplastic cells and other elements of the tumor microenvironment, supporting angiogenesis, fibrosis, and immunosuppression and sustaining the tumor growth and spread [16].

Taking into account the pathophysiology, pancreatic inflammatory disorders are interesting from the point of view of the regeneration process of injured tissue. Acute pancreatitis (AP) is a two-faced disease; mild/moderate AP (MAP) is a typically self-limiting disorder with good prognosis, whereas severe AP (SAP) represents an example of an inflammatory process with excessive production of cytokines leading to organ failure, need for intensive care, and patient death. Despite prognostic scales and some biochemical markers allowing the prediction of disease severity, there is still a need for reliable and feasible clinical markers to predict a severe disease course. In contrast to AP, chronic pancreatitis (CP) is a process related to irreversible morphological changes to the gland, which significantly increase the risk of pancreatic cancer development and can be treated as a premalignant condition [17].

Moreover, the regeneration of both the exocrine and endocrine parts of the pancreas is still an area of scientific debate [18].

Experimental studies in animal models indicate that BMSCs play an important role in pancreatic tissue regeneration, and transplantation of BMSCs during the course of AP can lead to clinical improvement resulting in pancreatic tissue healing [19–23]. This suggests that BMSCs may be used for the treatment of AP, especially severe AP, in the future [19].

In the current study, we analyzed the presence of select populations of BMSCs, including HSCs, EPCs, MSCs, and PSCs, such as very small embryonic-like stem cells (VSELs), in the PB of patients suffering from AP (mild or moderate/severe) or CP and subsequently correlated the absolute number of circulating BMSCs with the concentration of select chemoattractants. The experiments allowed us to verify whether the phenomenon of BMSC mobilization in pancreatic disorders is characteristic only for cancer patients, as presented during our previous study [12], or also occurs during AP or CP. We also found out the process of mobilization of some populations of cells is related to disease severity in AP.

## 2. Materials and Methods

### 2.1. Patients and Blood Samples

The study was approved by the Bioethical Committee of Pomeranian Medical University in Szczecin, Poland (approval no.: KB-0012/43/12). Patients enrolled in the current study were hospitalized in the Department of Gastroenterology of Pomeranian Medical University in Szczecin, Poland, in the years 2013-2018. PB samples (~30 mL) were collected from the forearm veins of 26 patients with CP, 26 patients with AP (*n* = 15 with mild AP (MAP) and *n* = 11 with moderate/severe AP (SAP)), and 26 healthy controls in the first 24-72 hours after hospital admission after receiving information from the physician and approving of an informed consent form. The diagnosis of AP and subdivision into MAP and SAP were based on criteria proposed in the 2012 revision of the Atlanta classification [24]. We established the diagnosis of CP based on the presence of the following typical morphological features within the pancreas: calcifications, dilation of the pancreatic duct, pancreatic duct stones, cysts and lobularity with honeycombing present on ultrasound, endoscopic ultrasound or abdominal computed tomography in patients with typical symptoms of CP, and history of chronic abuse of alcohol.

PB samples were centrifuged (370×*g*, 10 min) to separate serum. Serum samples were frozen and stored at -80°C until further assessment of select growth/inhibitory factors and immunomodulatory molecule concentrations. Red blood cells were removed from the PB samples using 1X hypotonic lysis solution (BD Pharm Lyse Buffer, BD Bioscience Pharmingen). Total nucleated cells (TNCs) were washed with Dulbecco's phosphate buffered saline (DPBS, w/o Ca^2+^ and Mg^2+^; HyClone, GE Healthcare Life Science). PB-derived TNCs were resuspended in staining buffer (DPBS supplemented with 2% fetal bovine serum (FBS), Merck) and immunolabeled with antibodies specific for VSELs, HSCs, EPCs, and MSCs. The phenotypes of the analyzed stem cell populations are given in Table 1.

### 2.2. Flow Cytometry

Circulating VSELs and HSCs were identified following immunofluorescent staining of PB-derived TNCs against CD45 (PE, clone: HI30, BD Bioscience), hematopoietic lineage markers (“Lin”), CD133/1 (APC, clone: AC133, Miltenyi Biotec), and CD34 (APC, clone: 581, BD Bioscience). FITC-conjugated murine antihuman hematopoietic lineage markers (“Lin”) included the following antibodies: anti-CD2 (clone: RPA-2.10), anti-CD3 (clone: UCHT1), anti-CD14 (clone: M*φ*P9), anti-CD16 (clone: 3G8), anti-CD19 (clone: HIB19), anti-CD24 (clone: ML5), anti-CD56 (clone: NCAM16.2), anti-CD66b (clone: G10F5), and anti-CD235a (clone: GA-R2; all antibodies from BD Bioscience). EPCs and CD133^+^SCs were identified following staining with monoclonal antibodies against CD45 (FITC, clone: HI30, BD Bioscience), CD133/1 (PE, clone: AC133), and CD31 (PE, clone: WM59, BioLegend). We used the following antibodies to label MSCs: anti-CD45 (FITC, clone: HI30), anti-CD105 (PE, clone: 43A3, BioLegend), and anti-Stro-1 (Alx647, clone: STRO-1, BioLegend). TNCs were incubated with the antibodies for 30 min on ice and washed with DPBS (w/o Ca^2+^ and Mg^2+^, HyClone). Subsequently, cells were fixed with 2% paraformaldehyde solution (Avantor Performance Materials Poland S.A.) for 15 min at room temperature and washed with DPBS (w/o Ca^2+^ and Mg^2+^, HyClone). Prior to analysis, cells were stained with Hoechst 33342 (2 *μ*g/mL, Merck) to distinguish nucleated cells from cell debris and analyzed by LSR II flow cytometry (Becton Dickinson). The absolute number of circulating stem cells was calculated per milliliter of PB.

### 2.3. ELISA

#### 2.3.1. Determination of the CXCL12/SDF-1 Concentration in Serum

The Human CXCL12/SDF-1 Immunoassay ELISA reagent kit (R&D Systems) was used to determine the serum CXCL12/SDF-1 alpha concentration. The determinations were made in accordance with the guidelines provided by the manufacturer. The CXCL12/SDF-1 alpha concentration was calculated from a previously prepared plate standard curve for each individual based on serial dilutions of the standard solution included in the commercial kit. The absorbance was measured on an EnVision microplate reader (Perkin Elmer) at 450 nm.

#### 2.3.2. Determination of the HGF Concentration in Serum

The human HGF immunoassay ELISA reagent kit (R&D Systems) was used to determine the serum HGF concentration. The determinations were made in accordance with the guidelines provided by the manufacturer. The HGF concentration was calculated from a previously prepared plate standard curve for each individual based on serial dilutions of the standard solution included in the commercial kit. The absorbance was measured on an EnVision microplate reader (Perkin Elmer) at 450 nm.

#### 2.3.3. Determination of C3a-desArg and C5a-desArg Concentrations in Serum

To determine the serum concentrations of C3adesArg and C5a-desArg, we used the Human C3a ELISA Kit and Human C5a ELISA Kit II (BD OptEIA™). These kits contain plates with wells coated with monoclonal antibodies specific for human C3a-desArg and C5a-desArg, respectively. The determinations were made in accordance with the guidelines provided by the manufacturer. The concentration of C3a-desArg and C5a-desArg was calculated from a previously prepared plate standard curve for each individual based on serial dilutions of the freeze-dried standard solution included in each commercial kit. The absorbance was measured on an EnVision microplate reader (Perkin Elmer) at 450 nm.

#### 2.3.4. Determination of the MAC Concentration in Serum

The Human C5b-9 ELISA Set (BD OptEIA™) was used to determine the serum MAC concentration. The determinations were made in accordance with the guidelines provided by the manufacturer. The MAC concentration was calculated from a previously prepared standard curve for each individual based on serial dilutions of the standard solution included in the commercial kit. The absorbance was measured on an EnVision microplate reader (Perkin Elmer) at 450 nm.

### 2.4. Reverse-Phase High-Performance Liquid Chromatography (RP-HPLC)

The serum and internal standard C17-S1P (Avanti Polar Lipids) used in this experiment were brought to room temperature. Plasma/serum (100 *μ*L) and 30 *μ*L of synthetic C17-S1P standard in methanol (MetOH):10 mM K2HPO4 (9 : 1, v/v; pH 7.2) were added to the glass tube. A mixture containing C17-S1P and C18-S1P was prepared under the same conditions. Samples were vortexed and 1 M NaCl added to obtain 1 mL. Subsequently, 1 mL of MetOH, 300 mL of concentrated HCl, and 2 mL of chloroform were added. Each step was preceded by mixing with a vortex. The samples were mixed on a test tube rotator for 20 min and centrifuged at 3500 rpm for 2 min at 20°C. The lower organic phase was withdrawn and transferred to a new tube. The upper layer was reextracted by adding 2 mL of chloroform, mixing on the test tube rotator for 10 min, and centrifuging again. The lower organic phase containing S1P was combined with the previous lower layer. The samples were then dried in a vacuum centrifuge (RVC 2–25 CD) at 45°C for 45–60 min. The dried extracts were stored at −80°C until analysis.

Before measurement, the extracts were brought to room temperature and dissolved in 130 *μ*L methanol and 20 *μ*L orthophthalaldehyde (OPA). Simultaneously, a mixture was prepared consisting of 30 *μ* C17-S1P, 30 *μ*L C18-S1P, and 940 *μ*L MetOH:K2HPO4 (9 : 1, v/v; pH 7.2), from which 600 *μ*L was taken, transferred to a new sample tube, and 75 *μ*L of OPA added. All samples with OPA were incubated for 20 min at room temperature in a dark room and then centrifuged at 6000 rpm for 10 min at 20°C. The supernatant was transferred to a new sample tube and 20 *μ*L of 10 mM K2HPO4 buffer (pH 7.2) added. After centrifugation, the mixture was immediately transferred to a clean bottle. Buffer samples were incubated for 10 min at 4°C and then centrifuged again (6000 rpm, 20°C, 10 min). After centrifugation, the clear supernatant was transferred to a clean bottle and RP-HPLC performed. Chromatographic data were developed using HP Chemstation software (Agilent, USA). A C18-ARII Cosmosil 5-*μ*m C18-ARII column (150 × 4.6) at 25°C and a 5-*μ*m C18-ARII precolumn (10 × 4.6, Waters) were used for separation in the reverse phase. An isocratic method with a mobile phase consisting of 10 mM K2HPO4 (pH 5.5) and methanol (15 : 85, v/v) was used. Samples (50 *μ*L) were injected on the column every 30 min at a flow rate of 1 mL/min. The wavelength for detecting S1P derivatives was 340 nm for excitation and 455 nm for emission. The S1P concentration was calculated on the basis of the peak surface area of the internal standard C17- S1P.

### 2.5. Statistical Analysis

As the distributions of the analyzed quantitative variables were significantly different from a normal distribution (Shapiro-Wilk's test), nonparametric tests were used, and medians with lower (Q1) and upper (Q3) quartiles were presented as descriptive statistics. The Mann–Whitney *U* test was performed to compare variables between groups. Correlations between parameters were assessed using Spearman's rank correlation coefficient (*r*). Statistical analysis was performed using Statistica 13 software. The significance level was defined as *P* < 0.05. Heatmaps were generated using OriginLab software.

## 3. Results

### 3.1. Analysis of Clinical and Biochemical Features of the Patients

We did not observe any significant differences between the groups of patients enrolled in the study when we compared gender and age, whereas the body mass index (BMI) was significantly lower in individuals with CP compared to other groups of patients. White blood cell count (WBC) was significantly higher in the patients with AP and CP compared to the healthy controls. A comparison of the general and clinical characteristics of the analyzed groups is presented in Table 2.

### 3.2. Analysis of BMSC Populations Circulating in the PB

**Figure 1:**
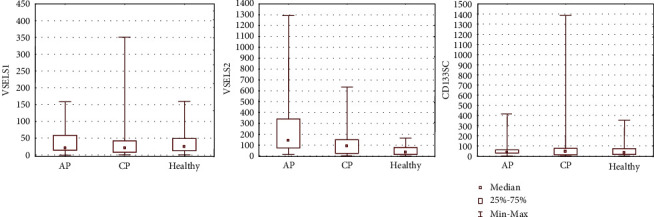
The absolute numbers of circulating cells from the VSELs“1”, VSELs“2”, and CD133^+^SC subpopulations in the PB of patients.

**Figure 2:**
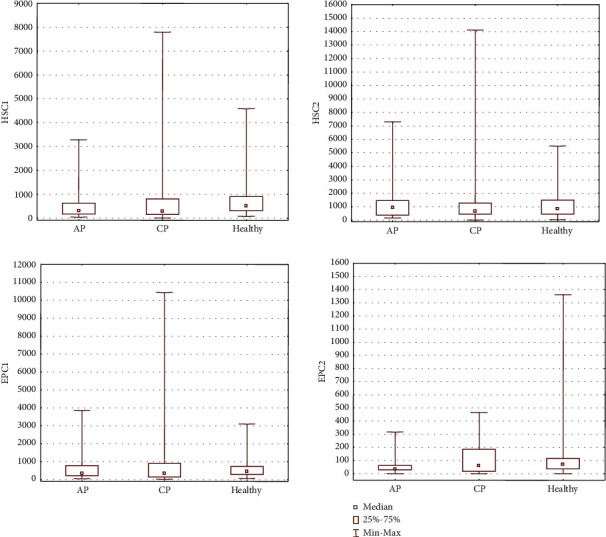
The absolute numbers of the HSCs“1”, HSCs“2”, EPCs“1”, EPCs“2” subpopulations circulating in the PB of patients.

**Figure 3:**
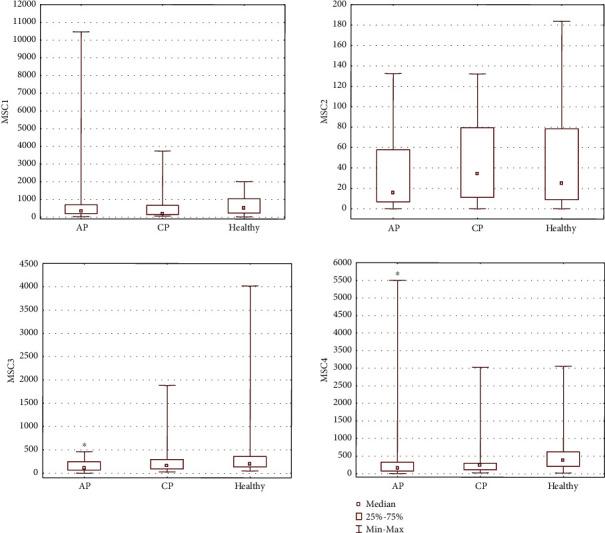
The absolute numbers of MSCs“1”, MSCs“2”, MSCs“3”, and MSCs“4” populations circulating in the peripheral blood of patients.

Flow cytometry revealed significantly higher absolute numbers of circulating PSCs, such as VSELs (identified as Lin^−^/CD45^−^/CD34^+^ subpopulation, VSELs“2”) in the PB in all groups of patients with AP (*P* = 0.000013), including MAP (*P* = 0.008) and SAP (*P* = 0.000006), as well as CP (*P* = 0.0009) compared to the control group. Interestingly, the number of circulating VSELs (identified as VSELs“2” subpopulation) was greater in AP than in CP (*P* = 0.06), suggesting an impact of acute inflammation on the mobilization process of these SCs (Figure 1, Table 3). The number of circulating VSELs (VSELs“2”) was also significantly higher in SAP than in MAP (*P* = 0.01). A slight increase in the total number of other circulating populations of BMSCs, such as EPCs“2”, HSCs“1”, HSCs“2”, and CD133^+^SCs, was also noted in AP patients compared to control individuals (Figure 2, Table 3). Moreover, we observed an increase in the absolute number of circulating EPCs (identified as EPCs“2”) and MSCs (identified as MSCs“2”) in CP groups, in parallel with an increase in the number of circulating CD133^+^SCs in CP compared to control individuals, though the difference was not significant (Figures 2 and 3, Table 3). A significant decrease in the number of circulating CD45^−^/STRO-1^+^/CD105^−^ (MSCs“3”, *P* = 0.003) and CD45^−^/CD90^+^/CD29^+^ (MSCs“4”, *P* = 0.003) MSCs was observed in AP (Figure 3, Table 3). We also observed a slight but nonsignificant decrease in the numbers of other MSC subpopulations (including MSCs“1” and MSCs“2”) and other nonhematopoietic BMSC populations (VSELs“1” and EPCs“1”) in AP patients compared to the healthy control group. In contrast, in CP patients, we observed a decreased absolute number of circulating MSC subpopulations (MSCs“1”, MSCs“3”, and MSCs“4”) and other BMSC populations (VSELs“1”, EPCs“1”, HSCs“1”, and HSCs“2”), though no significance was noted (Figures 1–3, Table 3). MAP patients also had a significantly decreased number of CD45^−^/CD90^+^/CD29^+^ MSCs (MSCs“4”, *P* = 0.003) compared to controls, whereas SAP patients exhibited decreased numbers of circulating CD45^−^/Stro-1^+^/CD105^−^ MSCs (MSCs“3”, *P* = 0.01) compared to controls (Figure 3, Table 3). Apart from the significant increase in the number of circulating VSELs“2” cells, a slight but nonsignificant increase in the numbers of VSELs“1”, HSCs“1”, HSCs“2”, EPCs“1”, and MSCs“4” cells was observed in the PB of SAP patients compared to MAP patients (Figures 1–3, Table 3).

To summarize the most prominent results, an increased number of circulating VSELs“2” was observed in AP and CP patients, which corresponded to a decreased number of circulating MSCs“1”, MSCs“3”, and MSCs“4” cells in both AP and CP compared to healthy individuals. Moreover, in the course of SAP, an increased total number of circulating VSELs, HSCs, EPCs“1”, and MSCs“4” were observed in the PB compared to MAP.

### 3.3. Concentrations of Chemoattractive Factors in Serum and their Potential Involvement in the Mobilization of BMSCs to the PB

**Figure 4:**
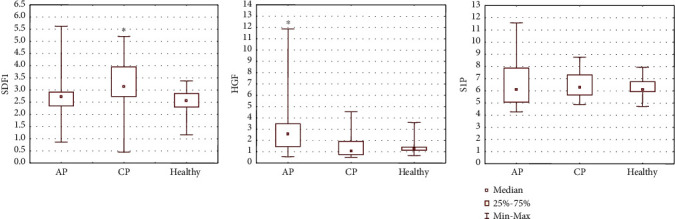
Serum concentrations of SDF1*α*, HGF, and S1P.

**Figure 5:**
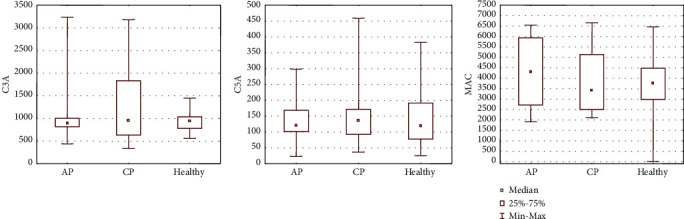
Serum concentrations of complement proteins C3a, C5a, and MAC.

In the next stage, we analyzed the concentrations of select chemoattractant molecules in the serum of patients enrolled in the current study and correlated them with the absolute number of cells within different BMSC populations circulating in the PB. We measured a significant elevation in SDF1*α* (*P* = 0.005) in the serum of CP patients and HGF (*P* = 0.002) in the serum of AP and MAP patients compared to healthy controls (Figure 4, Table 4). The lowest concentration of complement component C3a protein was measured in AP (both MAP and SAP) patients, and the concentration in CP patients was slightly lower than in healthy controls (Figure 5, Table 4). The concentration of C5a protein in serum was decreased in AP patients compared to healthy controls. The serum concentration of MAC complex was elevated in AP patients, especially in MAP, compared to the other groups of patients. In addition, a greater concentration of S1P was detected in MAP but remained at a similar level in the other groups of patients (Figure 5, Table 4).

**Figure 6:**
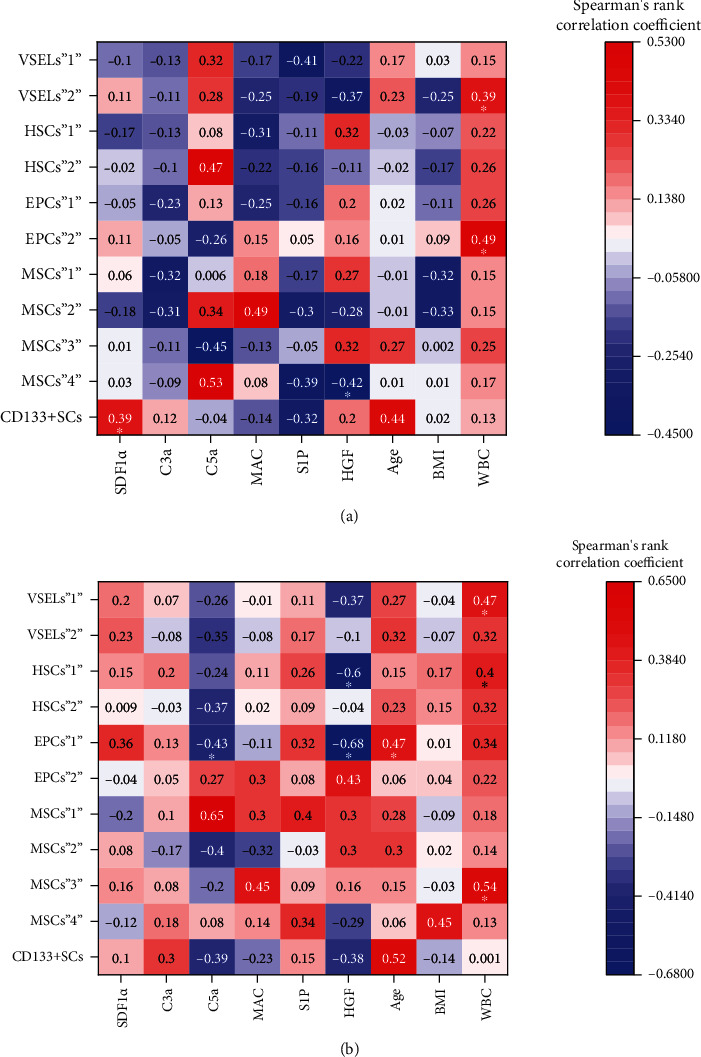
Heatmaps of the Spearman rank correlation coefficients between circulating populations of BMSCs and the concentration of chemoattractive factors or general patient characteristics. (a) AP group. (b) CP group. ∗*P* < 0.05.

**Figure 7:**
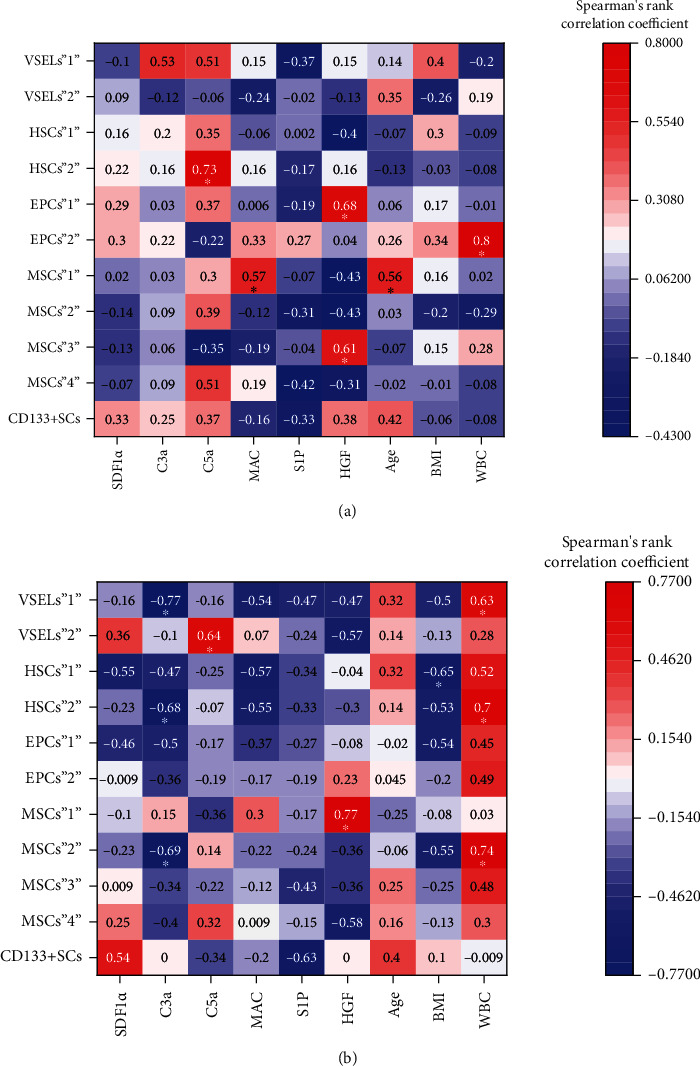
Heatmaps of the Spearman rank correlation coefficients between circulating populations of BMSCs and the concentration of chemoattractive factors or general patient characteristics. (a) MAP patients. (b) SAP patients. ∗*P* < 0.05.

Our analysis demonstrated both positive and negative correlations between the concentrations of the analyzed chemoattractive factors and the circulating BMSC subpopulations (Figures 6 and 7 and Supplementary Tables S1–S4). In AP patients, we observed positive correlations between the concentration of SDF1*α* and CD133^+^SC (identified as CD45^−^/CD31^−^/CD133^+^) mobilization (*r* = 0.39, *P* = 0.049), the concentration of C5a and HSCs“2” mobilization (*r* = 0.47, *P* = 0.017), the concentration of C5a and MSCs“4” mobilization (*r* = 0.53, *P* = 0.006), and the concentration of MAC and MSCs“2” mobilization (*r* = 0.49, *P* = 0.012), and negative correlations between the concentration of HGF and MSCs“4” mobilization (*r* = −0.43, *P* = 0.03), the concentration of S1P and VSELs“1” mobilization (*r* = −0.41, *P* = 0.04), and the concentration of C5a and MSCs“3” mobilization (*r* = −0.45, *P* = 0.023) (Figure 6(a), Supplementary Table S1). In CP patients, we observed positive correlations between the concentration of C5a and MSCs“1” mobilization (*r* = 0.64, *P* = 0.001), the concentration of MAC and MSCs“3” mobilization (*r* = 0.45, *P* = 0.05), and the concentration of HGF and EPCs“2” mobilization (*r* = 0.43, *P* = 0.001), and negative correlations between the concentration of C5a (*r* = −0.43, *P* = 0.043) or HGF (*r* = −0.68, *P* = 0.001) and EPCs“1” mobilization, and the concentration of HGF and HSCs“1” mobilization (*r* = −0.60, *P* = 0.06) (Figure 6(b), Supplementary Table S2). More positive correlations between chemoattractant concentrations and mobilized BMSC populations were observed in CP patients than AP patients (Figure 6, Supplementary Tables S1 and S2).

In MAP patients, we observed positive correlations between the concentration of C5a and HSCs“2” mobilization (*r* = 0.47, *P* = 0.017), the concentration of MAC and MSCs“1” mobilization (*r* = 0.56, *P* = 0.03), the concentration of HGF and EPCs“1” mobilization (*r* = 0.68, *P* = 0.007), the concentration of HGF and MSCs“3” mobilization (*r* = 0.61, *P* = 0.02), the concentration of C3a (*r* = 0.53, *P* = 0.05) or C5a (*r* = 0.51, *P* = 0.06) and VSELs“1” mobilization, and the concentration of C5a and MSCs“4” mobilization (*r* = 0.51, *P* = 0.05), and negative correlations between the concentration of S1P and VSELs“1” (*r* = −0.37, *P* = 0.19), MSCs“2” (*r* = −0.31, *P* = 0.26), MSCs“4” (*r* = −0.42, *P* = 0.27), or CD133^+^SC (*r* = −0.33, *P* = 0.25) mobilization and the concentration of HGF and HSCs“1” (*r* = −0.4, *P* = 0.16), MSCs“1” (*r* = −0.43, *P* = 0.12), MSCs“2” (*r* = −0.43, *P* = 0.12), or MSCs“4” (*r* = −0.31, *P* = 0.27) mobilization (Figure 7(a), Supplementary Table S3). In SAP patients, we observed positive correlations between the concentration of C5a and VSELs“2” mobilization (*r* = 0.64, *P* = 0.035), the concentration of HGF and MSCs“1” mobilization (*r* = 0.77, *P* = 0.005), and the concentration of SDF1*α* and CD133^+^SC (r = 0.54, *P* = 0.09) or VSELs“2” (*r* = 0.36, *P* = 0.27) mobilization, and negative correlations between the concentration of C3a and VSELs“1” (*r* = −0.77, *P* = 0.005), HSCs“1” (*r* = −0.47, *P* = 0.24), HSCs“2” (*r* = −0.68, *P* = 0.24), MSCs“2” (*r* = −0.69, *P* = 0.018), or EPCs“1” (*r* = −0.5, *P* = 0.11) mobilization; the concentration of SDF1*α* and HSCs“1” (*r* = −0.55, *p* = 0.07) or EPCs“1” (*r* = −0.46, *P* = 0.15) mobilization; the concentration of MAC and VSELs“1” (*r* = −0.54, *P* = 0.08), HSCs“1” (*r* = −0.57, *P* = 0.06), or HSCs“2” (*r* = −0.55, *P* = 0.07) mobilization; the concentration of S1P and VSELs“1” (*r* = −0.47, *P* = 0.14) or CD133^+^SC (*r* = −0.63, *P* = 0.03) mobilization; and the concentration of HGF and VSELs“1” (*r* = −0.47, *P* = 0.14), VSELs“2” (*r* = −0.57, *P* = 0.06), or MSCs“4” (*r* = −0.58, *P* = 0.06) mobilization (Figure 7(b), Supplementary Table S4).

Moreover, we observed some tendencies for generally positive correlations between the WBC and absolute numbers of circulating BMSCs in both AP (even more evident for most populations in SAP) and CP (Figure 6, Supplementary Tables S1 and S2). A positive correlation between age and absolute number of circulating BMSCs was observed in CP patients (Figures 6 and 7, Supplementary Tables S1–S4).

In summary, the presented results demonstrate that some populations of BMSCs, such as VSELs“2”, EPCs“2”, and CD133^+^SCs, are mobilized to the PB of patients with AP or CP. Interestingly, AP and CP patients had lower absolute numbers of MSCs (MSCs“1”, “3”, and “4”) in the PB compared to healthy controls. The phenomenon of SC mobilization to the PB is more evident in patients with SAP than MAP (in the case of VSELs, HSCs, MSCs“4”, and EPCs“1” subpopulations) and in patients with AP than CP (in the case of VSELs, HSCs, EPCs“2”, and MSCs“1” mobilization). We found that some innate immunity chemoattractive factors are significantly increased in the PB of patients with AP (i.e., HGF) and CP (i.e., SDF1*α*). We also noted a positive correlation between the C5a concentration and the mobilized population of VSELs“2” in both AP and SAP, between this molecule and the mobilized HSCs“2” population and between SDF1*α* and the mobilized population of CD133^+^SCs in AP. In MAP patients, the following significantly positive correlations were observed: between C5a concentration and mobilized population of HSCs“2” and HGF concentration and populations of EPCs“1”and MSCs“3” as well as MAC concentration and population of MSCs“1”. In general, more positive correlations between the concentrations of chemoattractive factors and the mobilization of select populations of BMSCs were observed for CP patients.

## 4. Discussion

Our study clearly shows that the SC mobilization to the PB is present in patients with AP or CP, though it is more evident in SAP. Various independent studies have shown that several populations of BMSCs, including VSELs, are mobilized to the PB of patients with different inflammatory disorders and diseases associated with tissue injury and regeneration [4–10]. The results of multiple clinical trials using BMSCs strongly suggest their important role in the process of tissue healing and regeneration [25, 26]. These observations led to further application of BMSCs in the treatment of some diseases, including systemic and local treatment of inflammatory bowel disease [27] or neurological and heart disorders [25, 28]. The phenomenon of BMSC mobilization and its role in tissues was also assessed in some oncological disorders, including animal studies on gastric cancer [14, 15], and in patients with pancreatic cancer [2, 12].

To the best of our knowledge, no data are available on the circulating levels of various populations of BMSCs in the PB of patients with pancreatic inflammatory disorders, and this phenomenon has not yet been examined in humans. Moreover, the issue of pancreatic regeneration is lively discussed in the scientific literature, with a focus on the endocrine pancreas and pancreatic islet regeneration and its meaning in the treatment of diabetes [18, 22, 29–34]. Dor et al. revealed that mature *β*-cells retain significant proliferative potential and their self-duplication capacity rather than SCs differentiation playing a major role in the process of *β*-cell replenishment [30]. This contradicts other studies in animal models of AP in which transplantation of some populations of BMSCs (mainly MSCs) led to a decrease in pancreatic amylase and inflammatory cytokines, as well as a reduction in pancreatic edema, necrosis, repair of intestinal mucosa, and improvement in the course of pancreatitis-associated lung injury [19, 20, 21, 35]. Furthermore, in the murine model of streptozocin-induced pancreatic damage, transplanted BM-derived cells engrafted in both the exocrine (ductal epithelium) and endocrine (islets sites) pancreas result in insulin production [33]. BMSCs differentiation into pancreatic stellate cells, key players in the complex pancreatic microenvironment, was also noted during the course of AP and CP [36]. There is growing evidence that VSELs also play a role in pancreas repair [22, 37–40]. The presence of a population of cells expressing VSEL characteristics in various murine organs, including the pancreas, was reported by other investigators [41–43]. Abouzaripour et al. showed, in streptozotocin-induced diabetes mice, that VSELs, administered into the tail vein, migrated to the pancreas and contributed to improving the glycemic profile and weight gain [37]. Bhartiya et al. reported an increased number of murine VSELs (small-sized Lin^−^/CD45^−^/Sca-1^+^ cells) in the remnant pancreas after pancreatectomy [39]. Their own observations and the unique biological properties of cells detected in smears of pancreatic tissue led Bhartiya et al. to draw the following conclusion about the role of VSELs in pancreatic endocrine and exocrine biology and regeneration: VSELs are mobilized in response to pancreatectomy, differentiate into pancreatic progenitors, and possibly regenerate both pancreatic islets and acinar cells [39]. However, it has been shown that type 2 diabetes occurs as a result of decreased *β*-cell function, possibly resulting from an age-related compromised niche that does not allow VSELs to maintain normal homeostasis [22, 39]. Our observations showing the process of mobilization of BMSCs in pancreatic inflammatory disorders may shed some light on the possible role of BMSCs in the process of pancreatic regeneration. Interestingly, the phenomenon of SC mobilization to the PB is more evident in patients with SAP than MAP and in patients with AP than CP. It should be underlined that SAP, in comparison to MAP, is related to significantly higher risk of organ failure, need for intensive care, and patient death. Predicting the course of the disease on admission is not easy for clinicians. There are some prognostic scales and biochemical markers allowing clinical prediction of disease severity [24, 44], but their lack of specificity, limited sensitivity, and complexity make them hard to apply in everyday clinical practice. Therefore, there is still a need for reliable and feasible markers allowing the prediction of a SAP. Taking into account, that the numbers of VSELs are significantly higher in patients with SAP than in MAP, their utility as a predictor of severe disease course seems to be an interesting direction for further research [45, 46].

The intensity of the inflammatory process, as measured using WBC, which is routinely used in clinical practice, and natural mobilization of the cells from the BM related to an inflammatory reaction, is natural but not the only one explanation of the phenomenon of mobilization.

We observed generally positive correlations between the WBC and absolute numbers of different BMSC populations in AP, CP, and SAP and significant positive correlations between the WBC and absolute number of mobilized subpopulations of VSELs in AP (including SAP) and CP. Experimental data also show that other innate immunity is involved in the process of SC migration [11]. Therefore, we aimed to check their impact on SCs mobilization in both AP and CP. According to the published data, SDF1*α* protein plays a crucial role in chemoattraction of the SCs [20, 47, 48], whereas *in vivo* studies have shown increased SDF1*α* expression in the inflamed pancreas and confirmed that migration of BMSCs to the pancreas positively correlates with SDF1*α* concentration [19, 20]. Other studies have indicated that the SDF1*α*/CXCR4 axis plays an important role in BMSC mobilization in other inflammatory conditions, such as myocardial ischemia [47], brain ischemia [48], wound healing [49], or AP [20]. In a rat model of AP, Gong et al. reported elevation of SDF1*α* in the injured pancreas and BMSC migration to the pancreatic parenchyma [20]. In agreement with this, we found that the concentration of SDF1*α* protein was significantly elevated in CP patients, with slight elevation observed in MAP patients. Moreover, SDF1*α* concentration positively correlated with CD133^+^SCs mobilization in AP patients and CD133^+^SCs and VSELs subpopulation mobilization in SAP patients. However, in contrast to CP, the correlations between SDF1*α* concentration and the absolute number of circulating BMSCs were negative or indifferent in AP patients (excluding CD133^+^SCs, VSELs“2”, and EPCs“2”). Our previous study on pancreatic cancer patients showed that significant mobilization of VSELs in patients with pancreatic cancer is related to elevated levels of complement cascade proteins (C5a and C5b-9/membrane attacking complex) and S1P, whereas elevated MSCs level positively correlates with HGF [12]. This is in line with our study showing that C5a may be involved in the mobilization of VSELs (identified as CD45^−^/Lin^−^/CD34^+^) in patients with SAP and AP, but not in CP patients. Interestingly, we observed opposite negative correlations between the C5a concentration and mobilization of some BMSC populations (e.g., EPCs“2” and MSCs“3”) in AP patients, as well as the C5a concentration and majority of circulating BMSC populations (except EPCs“2”, MSCs“1”, and MSCs“4”) in CP patients. Similarly, negative correlations between the S1P concentration and majority of BMSCs were observed in AP patients, whereas these correlations were positive for almost every population of BMSCs in CP patients.

These relationships show that, depending on the type of disease course (acute vs. chronic), other mechanisms may underlie the process of BMSC mobilization. This could be explained by the modulatory role of cytokines and the fact that AP is related to significant elevation of cytokine concentrations in PB and tissues (commonly called a “cytokine storm”) [50]. This process is similar to sepsis in terms of pathophysiology; therefore, it is no surprise that some populations of circulating BMSCs (including CD45^−^/Lin^−^/CD34^+^ VSELs) were previously found to be elevated in PB from patients with sepsis [6, 51]. Considering the above, we have to underline that immunomodulatory factors play a permissive role in severe inflammation. Previously, our team showed that elevated concentrations of some interleukins and TNF-*α* in the PB of pancreatic cancer patients were significantly associated with the number of circulating BM-derived MSCs [52]. Surprisingly, in contrast to our previous observations concerning the issue of BMSC circulation in the PB of patients with inflammatory and neoplastic disorders, our data show that the absolute numbers of all analyzed MSCs populations in AP patients, and the majority of these subpopulations in CP patients were decreased compared to the control group. The phenomenon of decreased numbers of circulating populations of MSCs may be explained by their fast migration from the BM to sites of inflammation. The targeted migration was previously described in a rat model of silica-induced pulmonary fibrosis, in which intravenously injected BM-derived MSCs migrated to the injured lungs, leading to the attenuation of pulmonary fibrosis [53]. Moreover, the study showed that the fluorescent signal originating from transplanted cells declines over time; the intensity of the fluorescence of engrafted cells was highest 6 h after injection and decreased sharply on the third day [53], indicating that the time of blood collection greatly influences the results. In the current study, blood was collected from the patients between the first and third day of the hospital stay, which may not correlate with the level of mobilization and engraftment of these cells. Moreover, recent evidence indicates that, in the murine model of sodium taurocholate-induced SAP, intravenous administration of BM-derived MSCs inhibited necrosis, repaired pancreatic injury, and reduced the systemic inflammatory response [54, 55]. It is important to underline that the presence of CD90 on the MSC surface (identified as the MSCs“4” subpopulation in the current study) is associated with higher growth and regenerative potential, whereas MSCs possessing Stro-1 antigen (also MSCs“4”), which is considered a marker of MSC multipotency, were able to generate colony forming units and multiple mesenchymal lineages and are responsible for immunosuppressive effects *in vitro* [56]. CD105 is considered as one of the markers of MSCs along with their capability of differentiating towards adipogenic, osteogenic, and chondrogenic lineages [56]; therefore, we identified subpopulations of cells (MSCs“1” or MSCs“2”) meeting also the criteria for MSC identification. Taking into account the aforementioned properties of these cells and the significant decrease in circulating MSCs“3” and MSCs“4” subpopulations in patients with AP, it is not surprising that MSCs expressing surface antigens Stro-1 and CD105 may be recruited into damaged tissue and play a role in pancreatic regeneration. These observations may also suggest that various subpopulations of MSCs with distinct properties and responsiveness on molecular signals may be present in BM, which needs to be further investigated.

The decreased number of circulating EPCs observed in our study in every subgroup of AP patients cannot be explained by older age or coexisting disorders. Vasa et al. reported that the number of circulating EPCs and their migratory capacity inversely correlate with coronary artery disease and hypertension [57], whereas Valgimigli et al. indicated that the number of circulating EPCs decreases in patients suffering from severe heart failure [58]. Interestingly, young mice with BM transplanted from old animals exhibit impaired neovascularization following corneal micropocket injury, indicating that the process of aging characterized by impaired neovascularization may be associated with dysfunctional EPCs and defective vasculogenesis [59].

In the current study, similarly to other studies, we noted significant elevation of the HGF concentration, in the PB of AP patients, which may serve as a chemotactic factor for MSCs or can be excreted by the MSCs [60–62]. Interestingly, elevated HGF may have clinical and prognostic value and allow prediction of the severe form of AP [63]. In our study we could observe some positive correlations between HGF and SCs concentrations. However, in contrary to previous observations, the levels of HGF were higher in mild form of AP comparing to severe; thus, we could not confirm its utility as a marker of severity.

## 5. Conclusions

Different populations of bone marrow cells including BMSCs are mobilized to the PB of patients with AP and CP. The obtained results suggest that some populations of BMSCs may play a role in pancreatic repair in pancreatitis and may be helpful in predicting a severe clinical course of the disease. The innate immune molecules are involved in the process of mobilization, and depending on the type of disease course, the other mechanisms of innate immunity may play a role in BMSC mobilization.

## Figures and Tables

**Table 1:** Analyzed BMSC populations and their corresponding phenotypes. EPC: endothelial progenitor cell; HSC: hematopoietic stem cell; MSC: mesenchymal stem/stromal cell; VSEL: very small embryonic-like stem cell.

Type of stem cell	Phenotype (name used in the study)
VSEL	CD45^−^/Lin^−^/CD133^+^ (VSELs“1”) CD45^−^/Lin^−^/CD34^+^ (VSELs“2”)

HSC	CD45^+^/Lin^−^/CD133^+^ (HSCs“1”) CD45^+^/Lin^−^/CD34^+^ (HSCs“2”)

EPC	CD45^−^/CD31^+^/CD133^+^ (EPCs“1”) CD45^−^/CD31^+^/CD34^+^/KDR^+^ (EPCs“2”)

MSC	CD45^−^/Stro-1^+^/CD105^+^ (MSCs“1”) CD45^−^/Stro-1^−^/CD105^+^ (MSCs“2”) CD45^−^/Stro-1^+^/CD105^−^ (MSCs“3”) CD45^−^/CD90^+^/CD29^+^ (MSCs“4”)

CD133^+^ stem cell	CD45^−^/CD31^−^/CD133^+^ (CD133^+^SCs)

**Table 2:** General and clinical characteristics of the patients enrolled in the study.

	CG	AP	Mild AP	Moderate/severe AP	CP
Number of patients	26	26	15	11	26
Sex, M/F	17/9	16/10	10/5	6/5	22/4
Age, years	49 (40-54.5)	46 (37.8-56.3)	46 (38-56)	48 (35-57)	49.5 (41-62)
BMI, kg/m^2^	24.9 (22.8-27.6)	25.9 (22.1-29.7)	25.8 (22.6-29.5)	25.9 (21.1-30.1)	22.7 (19.3-26)∗
WBC	6.55 (5.5-8.0)	10.8 (6.4-13.3)∗	9.42 (5.9-11.9)	12.9 (10.2-14.2)∗	7.4 (6.8-8.8)∗

AP: acute pancreatitis; BMI: body mass index; CP: chronic pancreatitis; CG: control group; WBC: white blood cells. Values are given as *N* or median (Q1-Q3). ∗*P* < 0.05 vs. healthy controls.

**Table 3:** The absolute numbers of subpopulations of BMSCs circulating in the peripheral blood of patients.

	VSELs“1”	VSELs“2”	HSCs“1”	HSCs“2”	EPCs“1”	EPCs“2”	MSCs“1”	MSCs“2”	MSCs“3”	MSCs“4”	CD133 + SCs
CG	38.7 (11.5-50.8)	45.2 (10.9-81.3)	509.8 (254.3-902.3)	793 (398.8-1414.7)	423.8 (269-753.8)	55.5 (23.3-113)	488.7 (176-805)	24.8 (8-74.2)	175 (108.1-268.6)	364.5 (155.7-612.6)	33.8 (11.2-74.8)
AP	36.9 (12.1-58.9)	*294.6*∗ (69.6-373.9)	540.6 (151.7-684.3)	1208 (347.5-1483.3)	324.5 (187.3-911.2)	63.2 (23.6-67.5)	350.8 (174.4-761.2)	15.6 (6-59.2)	*110.3*∗ (52.9-253.9	154.2∗ (59-340.3)	36 (25.3-63.2)
MAP	19.4 (10.8-39)	*82.8*∗ (37.3-194.3	254.7 (142.1-630.1)	822.1 (275-1467.3)	216.4 (139.6-670.2)	42.6 (25.5-78.2)	369.9 (174.8-720.9)	16.6 (4.3-57.2)	118.7 (36.5-280.3)	*78.2*∗ (41.3-317)	40 (25.4-64.6)
SAP	24.7 (12.4-72)	*336.9*∗ (11.5-877.8)	367 (215.2-859.6)	985.4 (574.9-1845.1)	363.8 (219.5-937.9)	29.8 (9.9-53.9)	311.8 (144.5-917.1)	12 (9.2-72.8)	*77.3*∗ (54.6-132.3)	235.8 (78.1-855.4)	32.2 (16.3-62.1)
CP	19.9 (6.8-42.5)	*90.4*∗ (18.4-155.2)	279.7 (132.8-846)	657.6 (415.3-1347.3)	327.5 (119.5-974.5)	57.9 (12.9-193)	203.9 (118.5-691.3)	34.3 (10.4-81.9)	161.7 (83.9-311.4)	233.9 (90.9-430.8)	44.6 (8.5-78.6)

AP: acute pancreatitis; CG: control group; CP: chronic pancreatitis; EPC: endothelial progenitor cell; HSC: hematopoietic stem cell; MAP: moderate acute pancreatitis; MSC: mesenchymal stem cell; SC: stem cell; SAP: severe acute pancreatitis; VSEL: very small embryonic-like stem cell. Values are presented as median (Q1-Q3). ∗*P* < 0.05 vs. healthy controls.

**Table 4:** The levels of chemoattractive factors in patients.

	SDF1*α* [ng/mL]	C3a [ng/mL]	C5a [ng/mL]	MAC [ng/mL]	S1P [nmol/mL]	HGF [ng/mL]
CG	2.6 (2.4-2.9)	992.3 (845.6-1103)	134.4 (77.3-189.3)	3412.6 (2486-5145)	6.0 (5.8-6.5)	1.3 (1.1-1.3)
AP	2.7 (2.3-3.0)	891.6 (771-1030.3)	120.2 (94.8-176.4)	4293.0 (2645.8-5951)	6.1 (4.8-8.2)	*2.6*∗ (1.3-3.6)
MAP	2.8 (2.3-3.0)	890.1 (791.4-1006.6)	114.3 (86.6-149.6)	4631.5 (2929.1-5974.3)	7.3 (4.6-8.8)	*2.7*∗ (1.7-3.3)
SAP	2.7 (2.3-3.3)	891.7 (731-1102)	137.3 (120.2-191.2)	3163.7 (2381.9-5019.6)	5.9 (5.0-6.9)	2.3 (0.9-5.2)
CP	*3.1*∗ (2.7-3.9)	953.3 (619.3-1881.8)	136.2 (90.5-183.3)	3408.0 (2758.7-4336.4)	6.3 (5.6-7.4)	1.0 (0.7-1.9)

AP: acute pancreatitis; C: complement; CG: control group; CP: chronic pancreatitis; HGF: hepatocyte growth factor; MAC: membrane attacking complex; MAP: moderate acute pancreatitis; S1P: sphingosine-1-phosphate; SAP: severe acute pancreatitis; SDF: stromal-derived factor 1*α*. Values are presented as median (Q1-Q3). ∗*P* < 0.05 vs. healthy controls.

## Data Availability

Data are found in the supplementary information files.

## Supplementary Materials

Supplementary Materials Table S1. The correlations between circulating populations of BMSCs and concentration of chemoattractive factors or general patients' characteristics in acute pancreatitis (AP) patients. EPCs: endothelial progenitor cells. HSCs: hematopoietic stem cells. MSCs: mesenchymal stem/stromal cells. SCs: stem cells. VSELs: very small embryonic-like stem cells. Spearman rank correlation coefficients with corresponding *p* values are presented. Table S2. The correlation between circulating populations of BMSCs and concentration of chemoattractive factors or general patients' characteristics in chronic pancreatitis (CP) patients. EPCs: endothelial progenitor cells. HSCs: hematopoietic stem cells. MSCs: mesenchymal stem/stromal cells. SCs: stem cells. VSELs: very small embryonic-like stem cells. Spearman rank correlation coefficients with corresponding *p* values are presented. Table S3. The correlation between circulating populations of BMSCs and concentration of chemoattractive factors or general patients' characteristics in mild acute pancreatitis (MAP) patients. EPCs: endothelial progenitor cells. HSCs: hematopoietic stem cells. MSCs: mesenchymal stem/stromal cells. SCs: stem cells. VSELs: very small embryonic-like stem cells. Spearman rank correlation coefficients with corresponding p-values are presented. Table S4. The correlation between circulating populations of BMSC and concentration of chemoattractive factors general patients' characteristics in moderate/severe acute pancreatitis patients. EPCs: endothelial progenitor cells. HSCs: hematopoietic stem cells. MSCs: mesenchymal stem/stromal cells. SCs: stem cells. VSELs: very small embryonic-like stem cells. Spearman rank correlation coefficients with corresponding *p* values are presented.
