# Supplementary material for: Evidence of Stem Cells Mobilization in the Blood of Patients with Pancreatitis: A Potential Link with Disease Severity
Source: Stem Cells Int. 2022 Jul 8;2022:5395248. doi: 10.1155/2022/5395248 (PMC9286984; doi:10.1155/2022/5395248)
Supplement: Supplementary Materials — Table S1. The correlations between circulating populations of BMSCs and concentration of chemoattractive factors or general patients' characteristics in acute pancreatitis (AP) patients. EPCs: endothelial progenitor cells. HSCs: hematopoietic stem cells. MSCs: mesenchymal stem/stromal cells. SCs: stem cells. VSELs: very small embryonic-like stem cells. Spearman rank correlation coefficients with corresponding p values are presented. Table S2. The correlation between circulating populations of BMSCs and concentration of chemoattractive factors or general patients' characteristics in chronic pancreatitis (CP) patients. EPCs: endothelial progenitor cells. HSCs: hematopoietic stem cells. MSCs: mesenchymal stem/stromal cells. SCs: stem cells. VSELs: very small embryonic-like stem cells. Spearman rank correlation coefficients with corresponding p values are presented. Table S3. The correlation between circulating populations of BMSCs and concentration of chemoattractive factors or general patients' characteristics in mild acute pancreatitis (MAP) patients. EPCs: endothelial progenitor cells. HSCs: hematopoietic stem cells. MSCs: mesenchymal stem/stromal cells. SCs: stem cells. VSELs: very small embryonic-like stem cells. Spearman rank correlation coefficients with corresponding p-values are presented. Table S4. The correlation between circulating populations of BMSC and concentration of chemoattractive factors general patients' characteristics in moderate/severe acute pancreatitis patients. EPCs: endothelial progenitor cells. HSCs: hematopoietic stem cells. MSCs: mesenchymal stem/stromal cells. SCs: stem cells. VSELs: very small embryonic-like stem cells. Spearman rank correlation coefficients with corresponding p values are presented. [file 5395248.f1.zip › BMSCs pancreatitis Supplement.docx]

**Supplementary materials**

Table S1. The correlations between circulating populations of BMSCs and concentration of chemoattractive factors or general patients characteristics in acute pancreatitis (AP) patients. EPCs: endothelial progenitor cells. HSCs: hematopoietic stem cells. MSCs: mesenchymal stem/ stromal cells. SCs: stem cells. VSELs: very small embryonic-like stem cells. Spearman rank correlation coefficients with corresponding p-values are presented.

|  | | **SDF1α** | **C3a** | **C5a** | **MAC** | **S1P** | **HGF** | **Age** | **BMI** | **WBC** |
| --- | --- | --- | --- | --- | --- | --- | --- | --- | --- | --- |
| VSELs | CD45-/Lin-/CD133+  VSELs“1” | -0.1  p=0.61 | -0.13  p=0.25 | 0.32  p=0.11 | -0.17  p=0.40 | -0.41  p=0.04 | -0.22  p=0.27 | 0.17  p=0.35 | 0.03  p=0.9 | 0.15  p=0.47 |
|  | CD45-/Lin-/CD34+  VSELs“2” | 0.11  p=0.58 | -0.11  p=0.57 | 0.28  p=0.16 | -0.25  p=0.22 | -0.19  p=0.35 | -0.37  p=0.06 | 0.23  p=0.24 | -0.25  p=0.21 | **0.39***  **p=0.04** |
| HSCs | CD45+/Lin-/CD133+  HSCs“1” | -0.17  p=0.42 | -0.13  p=0.53 | 0.08  p=0.7 | -0.31  p=0.12 | -0.11  p=0.6 | 0.32  p=0.11 | -0.03  p=0.86 | -0.07  p=0.7 | 0.22  p=0.26 |
|  | CD45+/Lin-/CD34+  HSCs“2” | -0.02  p=0.91 | -0.10  p=0.62 | 0.47  p=0.017 | -0.22  p=0.28 | -0.16  p=0.42 | -0.11  p=0.57 | -0.02  p=0.9 | -0.17  p=0.39 | 0.26  p=0.19 |
| EPCs | CD45-/CD31+/CD133+  EPCs“1” | -0.05  p=0.91 | -0.23  p=0.25 | 0.13  p=0.52 | -0.25  p=0.21 | -0.16  p=0.44 | 0.20  p=0.32 | 0.02  p=0.9 | -0.11  P=0.56 | 0.26  P=0.2 |
|  | CD45-/CD31+/CD34+/KDR+  EPCs“2” | 0.11  P=0.6 | -0.05  p=0.8 | -0.26  p=0.2 | 0.15  p=0.47 | 0.05  p=0.8 | 0.16  p=0.44 | 0.01  p=0.96 | 0.09  p=0.64 | **0.49***  **p=0.01** |
| MSCs | CD45-/Stro-1+/CD105+  MSCs“1” | 0.06  p=0.76 | -0.32  p=0.12 | 0.006  p=0.97 | 0.18  p=0.38 | -0.17  p=0.41 | 0.27  p=0.18 | -0.01  p=0.94 | -0.32  p=0.1 | 0.15  p=0.45 |
|  | CD45-/Stro-1-/CD105+  MSCs“2” | -0.18  p=0.4 | -0.31  p=0.12 | 0.34  p=0.09 | 0.49  p=0.012 | -0.3  p=0.16 | -0.28  p=0.16 | -0.01  p=0.94 | -0.33  p=0.1 | 0.15  p=0.45 |
|  | CD45-/Stro-1+/CD105-  MSCs“3” | 0.01  p=0.95 | -0.11  p=0.6 | -0.45  p=0.023 | -0.13  p=0.52 | -0.05  p=0.78 | 0.32  p=0.11 | 0.27  P=0.17 | 0.002  p=0.99 | 0.25  P=0.22 |
|  | CD45-/CD90+/CD29+  MSCs“4” | 0.03  p=0.87 | -0.09  p=0.68 | 0.53  p=0.006 | 0.08  p=0.7 | -0.39  p=0.05 | **-0.42***  **p=0.03** | 0.01  p=0.96 | 0.01  p=0.96 | 0.17  P=0.41 |
| CD133+ SCs | CD45-/CD31-/CD133+  CD133+ SCs | **0.39***  **p=0.049** | 0.12  p=0.56 | -0.04  p=0.85 | -0.14  p=0.5 | -0.32  p=0.11 | 0.2  p=0.33 | 0.44*  p=0.02 | 0.02  p=0.91 | 0.13  p=0.53 |

EPC: endothelial progenitor cell, HSC: hematopoietic stem cell, MSC: mesenchymal stem/stromal cell, SC: stem cell, VSEL: very small embryonic-like stem cell.

Spearman rank correlation coefficients with corresponding p-values are presented.

*P<0.05 vs. healthy controls.

Table S2. The correlation between circulating populations of BMSCs and concentration of chemoattractive factors or general patients characteristics in chronic pancreatitis (CP) patients. EPCs: endothelial progenitor cells. HSCs: hematopoietic stem cells. MSCs: mesenchymal stem/ stromal cells. SCs: stem cells. VSELs: very small embryonic-like stem cells. Spearman rank correlation coefficients with corresponding p-values are presented.

|  | | SDF1α | C3a | C5a | MAC | S1P | HGF | Age | BMI | WBC |
| --- | --- | --- | --- | --- | --- | --- | --- | --- | --- | --- |
| VSELs | CD45-/Lin-/CD133+  VSELs“1” | 0.2  p=0.38 | 0.07  p=0.74 | -0.26  p=0.23 | -0.01  p=0.96 | 0.11  p=0.61 | -0.37  p=0.12 | 0.27  p=0.17 | -0.04  p=0.84 | **0.47***  **p=0.01** |
|  | CD45-/Lin-/CD34+  VSELs“2” | 0.23  p=0.29 | -0.08  p=0.7 | -0.35  p=0.1 | -0.08  p=0.74 | 0.17  p=0.43 | -0.1  p=0.67 | 0.32  p=0.1 | -0.07  p=0.71 | 0.32  p=0.1 |
| HSCs | CD45+/Lin-/CD133+  HSCs“1” | 0.15  p=0.52 | 0.2  p=0.4 | -0.24  p=0.28 | 0.11  p=0.65 | 0.26  p=0.25 | **-0.60***  **p=0.06** | 0.15  p=0.45 | 0.17  p=0.4 | **0.40***  **p=0.04** |
|  | CD45+/Lin-/CD34+  HSCs“2” | 0.009  p=0.97 | -0.03  p=0.9 | -0.37  p=0.09 | 0.02  p=0.94 | 0.09  p=0.7 | -0.04  p=0.09 | 0.23  p=0.25 | 0.15  p=0.47 | 0.32  p=0.11 |
| EPCs | CD45-/CD31+/CD133+  EPCs“1” | 0.36  p=0.1 | 0.13  p=0.56 | **-0.43***  **p=0.043** | -0.11  p=0.63 | 0.32  p=0.16 | **-0.68***  **p=0.001** | **0.47***  **p=0.013** | 0.01  p=0.95 | 0.34  p=0.09 |
|  | CD45-/CD31+/CD34+/KDR+  EPCs“2” | -0.04  p=0.08 | 0.05  p=0.82 | 0.27  p=0.26 | 0.3  p=0.23 | 0.08  p=0.73 | 0.43  p=0.08 | 0.06  p=0.77 | 0.04  p=0.85 | 0.22  p=0.31 |
| MSCs | CD45-/Stro-1+/CD105+  MSCs“1” | -0.2  p=0.37 | 0.1  p=0.65 | 0.65*  p=0.001 | 0.3  p=0.22 | 0.4  p=0.07 | 0.3  p=0.22 | 0.28  p=0.16 | -0.09  p=0.63 | 0.18  p=0.38 |
|  | CD45-/Stro-1-/CD105+  MSCs“2” | 0.08  p=0.7 | -0.17  p=0.45 | -0.4  p=0.06 | -0.32  p=0.17 | -0.03  p=0.88 | 0.3  p=0.22 | 0.3  p=0.16 | 0.02  p=0.91 | 0.14  p=0.4 |
|  | CD45-/Stro-1+/CD105-  MSCs“3” | 0.16  p=0.49 | 0.08  p=0.7 | -0.2  p=0.41 | 0.45  p=0.05 | 0.09  p=0.7 | 0.16  p=0.52 | 0.15  p=0.45 | -0.03  p=0.9 | **0.54***  **p=0.03** |
|  | CD45-/CD90+/CD29+  MSCs“4” | -0.12  p=0.62 | 0.18  p=0.43 | 0.08  p=0.73 | 0.14  p=0.6 | 0.34  p=0.14 | -0.29  p=0.25 | 0.06  p=0.77 | **0.45***  **p=0.02** | 0.13  p=0.55 |
| CD133+ SCs | CD45-/CD31-/CD133+  CD133+ SCs | 0.1  p=0.63 | 0.3  p=0.19 | -0.39  p=0.07 | -0.23  p=0.34 | 0.15  p=0.5 | -0.38  p=0.1 | 0.52*  p=0.006 | -0.14  p=0.47 | 0.001  p=0.99 |

EPC: endothelial progenitor cell, HSC: hematopoietic stem cell, MSC: mesenchymal stem/stromal cell, SC: stem cell, VSEL: very small embryonic-like stem cell.

Spearman rank correlation coefficients with corresponding p-values are presented.

*P<0.05 vs. healthy controls.

Table S3. The correlation between circulating populations of BMSCs and concentration of chemoattractive factors or general patients characteristics in mild acute pancreatitis (MAP) patients. EPCs: endothelial progenitor cells. HSCs: hematopoietic stem cells. MSCs: mesenchymal stem/ stromal cells. SCs: stem cells. VSELs: very small embryonic-like stem cells. Spearman rank correlation coefficients with corresponding p-values are presented.

|  | | SDF1α | C3a | C5a | MAC | S1P | HGF | Age | BMI | WBC |
| --- | --- | --- | --- | --- | --- | --- | --- | --- | --- | --- |
| VSELs | CD45-/Lin-/CD133+  VSELs“1” | -0.1  p=0.95 | 0.53  p=0.05 | 0.51  p=0.06 | 0.15  p=0.62 | -0.37  p=0.2 | 0.15  p=0.6 | 0.14  p=0.6 | 0.4  p=0.14 | -0.2  p=0.47 |
|  | CD45-/Lin-/CD34+  VSELs“2” | 0.09  p=0.74 | -0.12  p=0.7 | -0.06  p=0.82 | -0.24  p=0.4 | -0.02  p=0.9 | -0.13  p=0.66 | 0.35  p=0.2 | -0.26  p=0.35 | 0.19  p=0.49 |
| HSCs | CD45+/Lin-/CD133+  HSCs“1” | 0.16  p=0.58 | 0.20  p=0.47 | 0.35  p=0.21 | -0.06  p=0.98 | 0.002  p=0.99 | -0.4  p=0.16 | -0.07  p=0.8 | 0.3  p=0.29 | -0.09  p=0.74 |
|  | CD45+/Lin-/CD34+  HSCs“2” | 0.22  p=0.45 | 0.16  p=0.6 | **0.73***  **p=0.003** | 0.16  p=0.58 | -0.17  P=0.56 | 0.16  p=0.58 | -0.13  p=0.64 | -0.03  p=0.9 | -0.08  p=0.77 |
| EPCs | CD45-/CD31+/CD133+  EPCs“1” | 0.29  p=0.32 | 0.03  p=0.89 | 0.37  p=0.19 | 0.006  p=98 | -0.19  p=0.5 | **0.68***  **p=0.007** | 0.06  p=0.82 | 0.17  p=0.53 | -0.01  p=0.97 |
|  | CD45-/CD31+/CD34+/KDR+  EPCs“2” | 0.3  p=0.3 | 0.22  p=0.45 | -0.22  p=0.45 | 0.33  p=0.25 | 0.27  p=0.34 | 0.04  p=0.9 | 0.26  p=0.34 | 0.34  p=0.21 | **0.8***  **p=0.0004** |
| MSCs | CD45-/Stro-1+/CD105+  MSCs“1” | 0.02  p=0.9 | 0.03  p=0.9 | 0.3  p=0.31 | **0.57***  **p=0.03** | -0.07  p=0.8 | -0.43  p=0.12 | **0.56***  **p=0.022** | 0.16  p=0.58 | 0.02  p=0.93 |
|  | CD45-/Stro-1-/CD105+  MSCs“2” | -0.14  p=0.63 | 0.09  p=0.73 | 0.39  p=0.16 | -0.12  p=0.7 | -0.31  p=0.27 | -0.43  p=0.12 | 0.03  p=0.9 | -0.2  p=0.43 | -0.29  p=0.29 |
|  | CD45-/Stro-1+/CD105-  MSCs“3” | -0.13  p=0.66 | 0.06  p=0.83 | -0.35  p=0.21 | -0.19  p=0.52 | -0.04  p=0.89 | **0.61***  **p=0.02** | -0.07  p=0.8 | 0.15  p=0.6 | 0.28  p=0.31 |
|  | CD45-/CD90+/CD29+  MSCs“4” | -0.07  p=0.82 | 0.09  p=0.73 | 0.51  p=0.06 | 0.19  p=0.5 | -0.42  p=0.13 | -0.31  p=0.27 | -0.02  p=0.92 | -0.01  p=0.95 | -0.08  p=0.76 |
| CD133+ SCs | CD45-/CD31-/CD133+  CD133+ stem cells | 0.33  p=0.25 | 0.25  p=0.39 | 0.37  p=0.24 | -0.16  p=0.6 | -0.33  p=0.25 | 0.38  p=0.18 | 0.42  p=0.12 | -0.06  p=0.83 | -0.08  p=0.78 |

EPC: endothelial progenitor cell, HSC: hematopoietic stem cell, MSC: mesenchymal stem/stromal cell, SC: stem cell, VSEL: very small embryonic-like stem cell.

Spearman rank correlation coefficients with corresponding p-values are presented.

*P<0.05 vs. healthy controls.

Table S4. The correlation between circulating populations of BMSC and concentration of chemoattractive factors general patients characteristics in moderate/severe acute pancreatitis patients. EPCs: endothelial progenitor cells. HSCs: hematopoietic stem cells. MSCs: mesenchymal stem/ stromal cells. SCs: stem cells. VSELs: very small embryonic-like stem cells. Spearman rank correlation coefficients with corresponding p-values are presented.

|  | | SDF1α | C3a | C5a | MAC | S1P | HGF | Age | BMI | WBC |
| --- | --- | --- | --- | --- | --- | --- | --- | --- | --- | --- |
| VSELs | CD45-/Lin-/CD133+  VSELs“1” | -0.16  p=0.63 | **-0.77***  **p=0.005** | -0.16  p=0.63 | -0.54  p=0.08 | -0.47  p=0.14 | -0.47  p=0.14 | 0.32  p=0.34 | -0.5  p=0.12 | **0.63***  **p=0.04** |
|  | CD45-/Lin-/CD34+  VSELs“2” | 0.36  p=0.27 | -0.1  p=0.77 | **0,64***  **p=0.035** | 0.07  p=0.83 | -0.24  p=0.47 | -0.57  p=0.06 | 0.14  p=0.67 | -0.13  p=0.69 | 0.28  p=0.4 |
| HSCs | CD45+/Lin-/CD133+  HSCs“1” | -0.55  p=0.07 | -0.47  p=0.24 | -0.25  p=0.47 | -0.57  p=0.06 | -0.34  p=0.39 | -0.04  p=0.91 | 0.32  p=0.34 | **-0.65***  **p=0.03** | 0.52  p=0.09 |
|  | CD45+/Lin-/CD34+  HSCs“2” | -0.23  p=0.48 | **-0.68***  **p=0.02** | -0.07  p=0.83 | -0.55  p=0.07 | -0.33  p=0.31 | -0.3  p=0.38 | 0.14  p=0.67 | -0.53  p=0.09 | **0.70***  **p=0.01** |
| EPCs | CD45-/CD31+/CD133+  EPCs“1” | -0.46  p=0.15 | -0.5  p=0.11 | -0.17  p=0.61 | -0.37  p=0.26 | -0.27  p=0.41 | -0.08  p=0.81 | -0.02  p=0.93 | -0.54  p=0.08 | 0.45  p=0.16 |
|  | CD45-/CD31+/CD34+/KDR+  EPCs“2” | -0.009  p=0.98 | -0.36  p=0.27 | -0.19  p=0.56 | -0.17  p=0.72 | -0.19  p=0.56 | 0.23  p=0.49 | 0.045  p=0.89 | -0.2  p=0.54 | 0.49  p=0.12 |
| MSCs | CD45-/Stro-1+/CD105+  MSCs“1” | -0.1  p=0.77 | 0.15  p=0.67 | -0.36  p=0.27 | 0.3  p=0.35 | -0.17  p=0.61 | **0.77***  **p=0.005** | -0.25  p=0.45 | -0.08  p=0.81 | 0.03  p=0.94 |
|  | CD45-/Stro-1-/CD105+  MSCs“2” | -0.23  p=0.5 | **-0.69***  **p=0.018** | 0.14  p=0.67 | -0.22  p=0.52 | -0.24  p=0.48 | -0.36  p=0.27 | -0.06  p=0.85 | -0.55  p=0.07 | **0.74***  **p=0.008** |
|  | CD45-/Stro-1+/CD105-  MSCs“3” | 0.009  p=0.98 | -0.34  p=0.3 | -0.22  p=0.52 | -0.12  p=0.73 | -0.43  p=0.19 | -0.36  p=0.27 | 0.25  p=0.47 | -0.25  p=0.45 | 0.48  p=0.13 |
|  | CD45-/CD90+/CD29+  MSCs“4” | 0.25  p=0.45 | -0.4  p=0.22 | 0.32  p=0.34 | 0.009  p=0.98 | -0.15  p=0.65 | -0.58  p=0.06 | 0.16  p=0.63 | -0.13  p=0.69 | 0.3  p=0.4 |
| CD133+ SCs | CD45-/CD31-/CD133+  CD133+ stem cells | 0.54  p=0.09 | 0  p=1 | -0.34  p=0.31 | -0.2  p=0.53 | -0.63*  p=0.03 | 0  p=1 | 0.4  p=0.23 | 0.1  p=0.75 | -0.009  p=0.98 |

EPC: endothelial progenitor cell, HSC: hematopoietic stem cell, MSC: mesenchymal stem/stromal cell, SC: stem cell, VSEL: very small embryonic-like stem cell.

Spearman rank correlation coefficients with corresponding p-values are presented.

*P<0.05 vs. healthy controls.
