# Supplementary material for: Evidence of Stem Cells Mobilization in the Blood of Patients with Pancreatitis: A Potential Link with Disease Severity
Source: Stem Cells Int. 2022 Jul 8;2022:5395248. doi: 10.1155/2022/5395248 (PMC9286984; doi:10.1155/2022/5395248)
Supplement: Supplementary Materials — Table S1. The correlations between circulating populations of BMSCs and concentration of chemoattractive factors or general patients' characteristics in acute pancreatitis (AP) patients. EPCs: endothelial progenitor cells. HSCs: hematopoietic stem cells. MSCs: mesenchymal stem/stromal cells. SCs: stem cells. VSELs: very small embryonic-like stem cells. Spearman rank correlation coefficients with corresponding p values are presented. Table S2. The correlation between circulating populations of BMSCs and concentration of chemoattractive factors or general patients' characteristics in chronic pancreatitis (CP) patients. EPCs: endothelial progenitor cells. HSCs: hematopoietic stem cells. MSCs: mesenchymal stem/stromal cells. SCs: stem cells. VSELs: very small embryonic-like stem cells. Spearman rank correlation coefficients with corresponding p values are presented. Table S3. The correlation between circulating populations of BMSCs and concentration of chemoattractive factors or general patients' characteristics in mild acute pancreatitis (MAP) patients. EPCs: endothelial progenitor cells. HSCs: hematopoietic stem cells. MSCs: mesenchymal stem/stromal cells. SCs: stem cells. VSELs: very small embryonic-like stem cells. Spearman rank correlation coefficients with corresponding p-values are presented. Table S4. The correlation between circulating populations of BMSC and concentration of chemoattractive factors general patients' characteristics in moderate/severe acute pancreatitis patients. EPCs: endothelial progenitor cells. HSCs: hematopoietic stem cells. MSCs: mesenchymal stem/stromal cells. SCs: stem cells. VSELs: very small embryonic-like stem cells. Spearman rank correlation coefficients with corresponding p values are presented. [file 5395248.f1.zip › SUpplementary BMSCs in AP and CP.pdf]

Supplementary materials

Table S1. **The correlations between circulating populations of BMSCs and concentration of chemoattractive factors or general patients characteristics in acute pancreatitis (AP) patients.** EPCs: endothelial progenitor cells. HSCs: hematopoietic stem cells. MSCs: mesenchymal stem/ stromal cells. SCs: stem cells. VSELs: very small embryonic-like stem cells.
Spearman rank correlation coefficients with corresponding p-values are presented.
*P<0.05 vs. healthy controls.

		**SDF1α**	**C3a**	**C5a**	**MAC**	**S1P**	**HGF**	**Age**	**BMI**	**WBC**
VSELs	CD45-/Lin-/CD133+ VSELs"1"	-0.1 p=0.61	-0.13 p=0.25	0.32 p=0.11	-0.17 p=0.40	-0.41 p=0.04	-0.22 p=0.27	0.17 p=0.35	0.03 p=0.9	0.15 p=0.47
	CD45-/Lin-/CD34+ VSELs"2"	0.11 p=0.58	-0.11 p=0.57	0.28 p=0.16	-0.25 p=0.22	-0.19 p=0.35	-0.37 p=0.06	0.23 p=0.24	-0.25 p=0.21	**0.39* p=0.04**
HSCs	CD45+/Lin-/CD133+ HSCs"1"	-0.17 p=0.42	-0.13 p=0.53	0.08 p=0.7	-0.31 p=0.12	-0.11 p=0.6	0.32 p=0.11	-0.03 p=0.86	-0.07 p=0.7	0.22 p=0.26
	CD45+/Lin-/CD34+ HSCs"2"	-0.02 p=0.91	-0.10 p=0.62	0.47 p=0.017	-0.22 p=0.28	-0.16 p=0.42	-0.11 p=0.57	-0.02 p=0.9	-0.17 p=0.39	0.26 p=0.19
EPCs	CD45-/CD31+/CD133+ EPCs"1"	-0.05 p=0.91	-0.23 p=0.25	0.13 p=0.52	-0.25 p=0.21	-0.16 p=0.44	0.20 p=0.32	0.02 p=0.9	-0.11 P=0.56	0.26 P=0.2
	CD45-/CD31+/CD34+/KDR+ EPCs"2"	0.11 P=0.6	-0.05 p=0.8	-0.26 p=0.2	0.15 p=0.47	0.05 p=0.8	0.16 p=0.44	0.01 p=0.96	0.09 p=0.64	**0.49* p=0.01**
MSCs	CD45-/Stro-1+/CD105+ MSCs"1"	0.06 p=0.76	-0.32 p=0.12	0.006 p=0.97	0.18 p=0.38	-0.17 p=0.41	0.27 p=0.18	-0.01 p=0.94	-0.32 p=0.1	0.15 p=0.45
	CD45-/Stro-1-/CD105+ MSCs"2"	-0.18 p=0.4	-0.31 p=0.12	0.34 p=0.09	0.49 p=0.012	-0.3 p=0.16	-0.28 p=0.16	-0.01 p=0.94	-0.33 p=0.1	0.15 p=0.45
	CD45-/Stro-1+/CD105- MSCs"3"	0.01 p=0.95	-0.11 p=0.6	-0.45 p=0.023	-0.13 p=0.52	-0.05 p=0.78	0.32 p=0.11	0.27 P=0.17	0.002 p=0.99	0.25 P=0.22
	CD45-/CD90+/CD29+ MSCs"4"	0.03 p=0.87	-0.09 p=0.68	0.53 p=0.006	0.08 p=0.7	-0.39 p=0.05	**-0.42* p=0.03**	0.01 p=0.96	0.01 p=0.96	0.17 P=0.41
CD133+ SCs	CD45-/CD31-/CD133+ CD133+ SCs	**0.39* p=0.049**	0.12 p=0.56	-0.04 p=0.85	-0.14 p=0.5	-0.32 p=0.11	0.2 p=0.33	0.44* p=0.02	0.02 p=0.91	0.13 p=0.53

Table S2. **The correlation between circulating populations of BMSCs and concentration of chemoattractive factors or general patients characteristics in chronic pancreatitis (CP) patients.** EPCs: endothelial progenitor cells. HSCs: hematopoietic stem cells. MSCs: mesenchymal stem/ stromal cells. SCs: stem cells. VSELs: very small embryonic-like stem cells.

Spearman rank correlation coefficients with corresponding p-values are presented.

*P<0.05 vs. healthy controls.

		SDF1α	C3a	C5a	MAC	S1P	HGF	Age	BMI	WBC
VSELs	CD45-/Lin-/CD133+ VSELs"1"	0.2 p=0.38	0.07 p=0.74	-0.26 p=0.23	-0.01 p=0.96	0.11 p=0.61	-0.37 p=0.12	0.27 p=0.17	-0.04 p=0.84	**0.47* p=0.01**
	CD45-/Lin-/CD34+ VSELs"2"	0.23 p=0.29	-0.08 p=0.7	-0.35 p=0.1	-0.08 p=0.74	0.17 p=0.43	-0.1 p=0.67	0.32 p=0.1	-0.07 p=0.71	0.32 p=0.1
HSCs	CD45+/Lin-/CD133+ HSCs"1"	0.15 p=0.52	0.2 p=0.4	-0.24 p=0.28	0.11 p=0.65	0.26 p=0.25	**-0.60* p=0.06**	0.15 p=0.45	0.17 p=0.4	**0.40* p=0.04**
	CD45+/Lin-/CD34+ HSCs"2"	0.009 p=0.97	-0.03 p=0.9	-0.37 p=0.09	0.02 p=0.94	0.09 p=0.7	-0.04 p=0.09	0.23 p=0.25	0.15 p=0.47	0.32 p=0.11
EPCs	CD45-/CD31+/CD133+ EPCs"1"	0.36 p=0.1	0.13 p=0.56	**-0.43* p=0.043**	-0.11 p=0.63	0.32 p=0.16	**-0.68* p=0.001**	**0.47* p=0.013**	0.01 p=0.95	0.34 p=0.09
	CD45-/CD31+/CD34+/KDR+ EPCs"2"	-0.04 p=0.08	0.05 p=0.82	0.27 p=0.26	0.3 p=0.23	0.08 p=0.73	0.43 p=0.08	0.06 p=0.77	0.04 p=0.85	0.22 p=0.31
MSCs	CD45-/Stro-1+/CD105+ MSCs"1"	-0.2 p=0.37	0.1 p=0.65	0.65* p=0.001	0.3 p=0.22	0.4 p=0.07	0.3 p=0.22	0.28 p=0.16	-0.09 p=0.63	0.18 p=0.38
	CD45-/Stro-1-/CD105+ MSCs"2"	0.08 p=0.7	-0.17 p=0.45	-0.4 p=0.06	-0.32 p=0.17	-0.03 p=0.88	0.3 p=0.22	0.3 p=0.16	0.02 p=0.91	0.14 p=0.4
	CD45-/Stro-1+/CD105- MSCs"3"	0.16 p=0.49	0.08 p=0.7	-0.2 p=0.41	0.45 p=0.05	0.09 p=0.7	0.16 p=0.52	0.15 p=0.45	-0.03 p=0.9	**0.54* p=0.03**
	CD45-/CD90+/CD29+ MSCs"4"	-0.12 p=0.62	0.18 p=0.43	0.08 p=0.73	0.14 p=0.6	0.34 p=0.14	-0.29 p=0.25	0.06 p=0.77	**0.45* p=0.02**	0.13 p=0.55
CD133+ SCs	CD45-/CD31-/CD133+ CD133+ SCs	0.1 p=0.63	0.3 p=0.19	-0.39 p=0.07	-0.23 p=0.34	0.15 p=0.5	-0.38 p=0.1	0.52* p=0.006	-0.14 p=0.47	0.001 p=0.99

Table S3. **The correlation between circulating populations of BMSCs and concentration of chemoattractive factors or general patients characteristics in mild acute pancreatitis (MAP) patients.** EPCs: endothelial progenitor cells. HSCs: hematopoietic stem cells. MSCs: mesenchymal stem/ stromal cells. SCs: stem cells. VSELs: very small embryonic-like stem cells.
Spearman rank correlation coefficients with corresponding p-values are presented.
*P<0.05 vs. healthy controls.

		SDF1α	C3a	C5a	MAC	S1P	HGF	Age	BMI	WBC
VSELs	CD45-/Lin-/CD133+ VSELs"1"	-0.1 p=0.95	0.53 p=0.05	0.51 p=0.06	0.15 p=0.62	-0.37 p=0.2	0.15 p=0.6	0.14 p=0.6	0.4 p=0.14	-0.2 p=0.47
	CD45-/Lin-/CD34+ VSELs"2"	0.09 p=0.74	-0.12 p=0.7	-0.06 p=0.82	-0.24 p=0.4	-0.02 p=0.9	-0.13 p=0.66	0.35 p=0.2	-0.26 p=0.35	0.19 p=0.49
HSCs	CD45+/Lin-/CD133+ HSCs"1"	0.16 p=0.58	0.20 p=0.47	0.35 p=0.21	-0.06 p=0.98	0.002 p=0.99	-0.4 p=0.16	-0.07 p=0.8	0.3 p=0.29	-0.09 p=0.74
	CD45+/Lin-/CD34+ HSCs"2"	0.22 p=0.45	0.16 p=0.6	**0.73* p=0.003**	0.16 p=0.58	-0.17 P=0.56	0.16 p=0.58	-0.13 p=0.64	-0.03 p=0.9	-0.08 p=0.77
EPCs	CD45-/CD31+/CD133+ EPCs"1"	0.29 p=0.32	0.03 p=0.89	0.37 p=0.19	0.006 p=98	-0.19 p=0.5	**0.68* p=0.007**	0.06 p=0.82	0.17 p=0.53	-0.01 p=0.97
	CD45-/CD31+/CD34+/KDR+ EPCs"2"	0.3 p=0.3	0.22 p=0.45	-0.22 p=0.45	0.33 p=0.25	0.27 p=0.34	0.04 p=0.9	0.26 p=0.34	0.34 p=0.21	**0.8* p=0.0004**
MSCs	CD45-/Stro-1+/CD105+ MSCs"1"	0.02 p=0.9	0.03 p=0.9	0.3 p=0.31	**0.57* p=0.03**	-0.07 p=0.8	-0.43 p=0.12	**0.56* p=0.022**	0.16 p=0.58	0.02 p=0.93
	CD45-/Stro-1-/CD105+ MSCs"2"	-0.14 p=0.63	0.09 p=0.73	0.39 p=0.16	-0.12 p=0.7	-0.31 p=0.27	-0.43 p=0.12	0.03 p=0.9	-0.2 p=0.43	-0.29 p=0.29
	CD45-/Stro-1+/CD105- MSCs"3"	-0.13 p=0.66	0.06 p=0.83	-0.35 p=0.21	-0.19 p=0.52	-0.04 p=0.89	**0.61* p=0.02**	-0.07 p=0.8	0.15 p=0.6	0.28 p=0.31
	CD45-/CD90+/CD29+ MSCs"4"	-0.07 p=0.82	0.09 p=0.73	0.51 p=0.06	0.19 p=0.5	-0.42 p=0.13	-0.31 p=0.27	-0.02 p=0.92	-0.01 p=0.95	-0.08 p=0.76
CD133+ SCs	CD45-/CD31-/CD133+ CD133+ stem cells	0.33 p=0.25	0.25 p=0.39	0.37 p=0.24	-0.16 p=0.6	-0.33 p=0.25	0.38 p=0.18	0.42 p=0.12	-0.06 p=0.83	-0.08 p=0.78

EPC: endothelial progenitor cells. HSCs: hematopoietic stem cells. MSCs: mesenchymal stem/ stromal cells. SCs: stem cells. VSELs: very small embryonic-like stem cells.
Spearman rank correlation coefficients with corresponding p-values are presented.
*P<0.05 vs. healthy controls.

Table S4. **The correlation between circulating populations of BMSC and concentration of chemoattractive factors general patients characteristics in moderate/severe acute pancreatitis patients.** EPCs: endothelial progenitor cells. HSCs: hematopoietic stem cells. MSCs: mesenchymal stem/ stromal cells. SCs: stem cells. VSELs: very small embryonic-like stem cells.
Spearman rank correlation coefficients with corresponding p-values are presented.
*P<0.05 vs. healthy controls.

		SDF1α	C3a	C5a	MAC	S1P	HGF	Age	BMI	WBC
VSELs	CD45-/Lin-/CD133+ VSELs"1"	-0.16 p=0.63	**-0.77* p=0.005**	-0.16 p=0.63	-0.54 p=0.08	-0.47 p=0.14	-0.47 p=0.14	0.32 p=0.34	-0.5 p=0.12	**0.63* p=0.04**
	CD45-/Lin-/CD34+ VSELs"2"	0.36 p=0.27	-0.1 p=0.77	**0,64* p=0.035**	0.07 p=0.83	-0.24 p=0.47	-0.57 p=0.06	0.14 p=0.67	-0.13 p=0.69	0.28 p=0.4
HSCs	CD45+/Lin-/CD133+ HSCs"1"	-0.55 p=0.07	-0.47 p=0.24	-0.25 p=0.47	-0.57 p=0.06	-0.34 p=0.39	-0.04 p=0.91	0.32 p=0.34	**-0.65* p=0.03**	0.52 p=0.09
	CD45+/Lin-/CD34+ HSCs"2"	-0.23 p=0.48	**-0.68* p=0.02**	-0.07 p=0.83	-0.55 p=0.07	-0.33 p=0.31	-0.3 p=0.38	0.14 p=0.67	-0.53 p=0.09	**0.70* p=0.01**
EPCs	CD45-/CD31+/CD133+ EPCs"1"	-0.46 p=0.15	-0.5 p=0.11	-0.17 p=0.61	-0.37 p=0.26	-0.27 p=0.41	-0.08 p=0.81	-0.02 p=0.93	-0.54 p=0.08	0.45 p=0.16
	CD45-/CD31+/CD34+/KDR+ EPCs"2"	-0.009 p=0.98	-0.36 p=0.27	-0.19 p=0.56	-0.17 p=0.72	-0.19 p=0.56	0.23 p=0.49	0.045 p=0.89	-0.2 p=0.54	0.49 p=0.12
MSCs	CD45-/Stro-1+/CD105+ MSCs"1"	-0.1 p=0.77	0.15 p=0.67	-0.36 p=0.27	0.3 p=0.35	-0.17 p=0.61	**0.77* p=0.005**	-0.25 p=0.45	-0.08 p=0.81	0.03 p=0.94
	CD45-/Stro-1-/CD105+ MSCs"2"	-0.23 p=0.5	**-0.69* p=0.018**	0.14 p=0.67	-0.22 p=0.52	-0.24 p=0.48	-0.36 p=0.27	-0.06 p=0.85	-0.55 p=0.07	**0.74* p=0.008**
	CD45-/Stro-1+/CD105- MSCs"3"	0.009 p=0.98	-0.34 p=0.3	-0.22 p=0.52	-0.12 p=0.73	-0.43 p=0.19	-0.36 p=0.27	0.25 p=0.47	-0.25 p=0.45	0.48 p=0.13
	CD45-/CD90+/CD29+ MSCs"4"	0.25 p=0.45	-0.4 p=0.22	0.32 p=0.34	0.009 p=0.98	-0.15 p=0.65	-0.58 p=0.06	0.16 p=0.63	-0.13 p=0.69	0.3 p=0.4
CD133+ SCs	CD45-/CD31-/CD133+ CD133+ stem cells	0.54 p=0.09	0 p=1	-0.34 p=0.31	-0.2 p=0.53	-0.63* p=0.03	0 p=1	0.4 p=0.23	0.1 p=0.75	-0.009 p=0.98
